# An observational study of febrile seizures: the importance of viral infection and immunization

**DOI:** 10.1186/s12887-016-0740-5

**Published:** 2016-12-03

**Authors:** Joshua R. Francis, Peter Richmond, Christine Robins, Katie Lindsay, Avram Levy, Paul V. Effler, Meredith Borland, Christopher C. Blyth

**Affiliations:** 1 Menzies School of Health Research, Charles Darwin University, Darwin, NT Australia; 2 Department of Paediatrics, Royal Darwin Hospital, Darwin, NT Australia; 3 Department of General Paediatrics, Princess Margaret Hospital, Perth, WA Australia; 4 School of Paediatrics and Child Health, University of Western Australia, Perth, WA Australia; 5 Wesfarmers Centre for Vaccines and Infectious Diseases, Telethon Kids Institute, University of Western Australia, Perth, WA Australia; 6 PathWest Laboratory Medicine, Princess Margaret Hospital, Perth, WA Australia; 7 PathWest Laboratory Medicine, QEII Medical Centre, Perth, WA Australia; 8 School of Pathology and Laboratory Medicine, University of Western Australia, Perth, WA Australia; 9 Communicable Disease Control, Department of Health, Perth, WA Australia; 10 Emergency Department, Princess Margaret Hospital, Perth, WA Australia; 11 School of Primary, Rural and Aboriginal Health Care, University of Western Australia, Perth, WA Australia; 12 Department of Infectious Diseases, Princess Margaret Hospital, Perth, WA Australia

**Keywords:** Febrile seizures, Immunization, Influenza, Enterovirus, Adenovirus

## Abstract

**Background:**

Febrile seizures are common in young children. Annual peaks in incidence mirror increased respiratory virus activity during winter. Limited virological data are available using modern diagnostic techniques for children with febrile seizures. We aimed to determine the frequency of detection of specific viral pathogens in children with febrile seizures, to describe risk factors including recent vaccination and clinical features associated with specific etiologies.

**Methods:**

An observational study was performed. Children aged 6 months to 5 years presenting to the Emergency Department of a tertiary children’s hospital in Western Australia with febrile seizures were enrolled between March 2012 and October 2013. Demographic, clinical data and vaccination history were collected, and virological testing was performed on per-nasal and per-rectal samples.

**Results:**

One hundred fifty one patients (72 female; median age 1.7y; range 6 m-4y9m) were enrolled. Virological testing was completed for 143/151 (95%). At least one virus was detected in 102/143 patients (71%). The most commonly identified were rhinoviruses (31/143, 22%), adenovirus (30/151, 21%), enteroviruses, (28/143, 20%), influenza (19/143, 13%) and HHV6 (17/143, 12%). More than one virus was found in 48/143 (34%). No significant clinical differences were observed when children with a pathogen identified were compared with those with no pathogen detected. Febrile seizures occurred within 14 days of vaccine administration in 16/151 (11%).

**Conclusion:**

At least one virus was detected in over two thirds of cases tested (commonly picornaviruses, adenovirus and influenza). Viral co-infections were frequently identified. Febrile seizures occurred infrequently following immunization.

## Background

Febrile seizures are common in children aged 6 months to 5 years, affecting 2–8% of children [1, 2]. Complex febrile seizures, featuring any of: focal seizures, prolonged seizure duration (greater than 15 min) or multiple febrile seizures in the course of a febrile illness, comprise 10–35% of all cases [1]. Simple febrile seizures are usually self-limiting. Neurological sequelae rarely result from either simple or complex febrile seizures [1, 2].

Seasonal variation in febrile seizures is observed and follows a similar pattern to that seen with common respiratory pathogens [3]. Despite this, there are limited data using modern diagnostic techniques examining the role of viral infections in children who present with febrile seizures [4, 5]. Previous studies have suggested a possible role for Influenza, Adenovirus and Human Herpesvirus 6 (HHV6) [4–9]. Recent literature has focused on the possible association between febrile seizures and complications of vaccine administration, with particular emphasis on Measles-containing vaccines [10–16]. In Australia in 2010 there was an increase of reported incidence of children with febrile adverse events following administration of one brand of trivalent influenza vaccine (bioCSL FluVax®). A 2011 report identified febrile seizures in 20% of 190 children presenting with vaccine-related febrile adverse events [17].

The Febrile seizures: Viruses and their Etiologic Role (FEVER) study was established to determine the frequency of detection of specific viral pathogens in children with febrile seizures, to describe risk factors and clinical features associated with specific pathogens, and to examine the role of recent immunizations in children presenting with febrile seizures.

## Methods

The FEVER study prospectively enrolled children aged 6 months to 5 years presenting to Princess Margaret Hospital Emergency Department (ED), the sole tertiary children’s hospital in Western Australia (WA). Patients were recruited between March 2012 and October 2013. Children were recruited on the basis of a history of seizure activity [18] within the context of a febrile illness which was defined as a history of fever in the preceding 96 h or a measured temperature of more than 37.5° Celsius whilst in the ED. A simple febrile seizure was defined as a single seizure of total duration 15 min or less with no focal features and complete neurological recovery. Complex febrile seizures featured any of: focal seizure activity, seizures lasting more than 15 min, multiple febrile seizures during the one febrile illness or incomplete neurological recovery. Children diagnosed with a central nervous system infection or encephalopathy were excluded from the study, as were those with a history of previous afebrile seizures or an underlying neurological disorder.

Following written consent from the parent/caregiver, demographics, immunization history and clinical data were collected. Results of all investigations carried out in the course of clinical care were recorded. Per-nasal samples were collected by trained healthcare staff using nasopharyngeal aspirate (NPA) or nasopharyngeal FLOQSwab™ (Copan Diagnostics Inc. Murrieta, CA). Stool or per-rectal swabs were collected where possible by trained healthcare staff and placed in viral transport media immediately after collection then transferred to the laboratory.

Nucleic acid was purified from clinical sample using the MagMAX Express-96 platform (Applied Biosystems) as previously described [19]. Respiratory samples underwent tandem multiplex real-time polymerase chain reaction (PCR) testing for respiratory viruses including Human Adenovirus species B-E; Human Bocavirus (HBoV); Human Coronaviruses OC43, 229E, HKU1 and NL63; Influenza viruses A and B; Parainfluenzaviruses 1–4; Human Metapneumovirus (HMPV) and Respiratory Syncytial Virus (RSV) types A and B [20]. Additional PCR assays were directed at the 5′UTR of Enteroviruses [21, 22]. Enterovirus species A-E and rhinovirus species A-C were identified by Sanger sequencing of the 5′UTR product. Per-rectal samples were tested using the same Enterovirus PCRs as well as a Rotavirus PCR [23].

Statistical analysis was performed using Microsoft Excel and GraphPad Prism 6.0 statistical software (GraphPad). Continuous variables were compared using the Student’s *t* test, and categorical variables were compared using Fisher’s exact test. A two-tailed *p* value of less than 0.05 was considered significant. Subjects with missing data were excluded from descriptive analyses for individual variables where data were missing. Ethical approval for the study was granted by the Human Research Ethics Committee at the Princess Margaret Hospital for Children in Perth, Australia (HREC number 1950/EP).

## Results

**Fig. 1:**
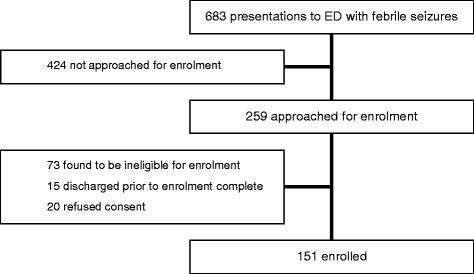
Enrolment flow chart

During the study period, 683 presentations to the ED were recorded to have had febrile seizures. Of these, 259 (38%) were approached for enrollment in the FEVER study. Seventy-three were found to be ineligible due to age, history of afebrile seizures or underlying neurological disorder, diagnosis of acute infection involving the central nervous system. Fifteen were eligible but not recruited prior to discharge, and consent was refused for 20. A total of 151 were enrolled in the study (Fig. 1). Children requiring admission were overrepresented in the cohort compared to those not enrolled (32% in the FEVER cohort vs 18% in all febrile seizures; *p* < 0.001).

Median age of enrolled patients was 1.7 years (range 6 months-4 years 9 months); 79/151 (52%) were male. A past history of febrile seizures was identified in 38/151 (25%) and a family of history of either febrile and/or afebrile seizures was identified in 46/151 (30%) and 24/151 (16%) respectively.

Median duration of symptoms prior to presentation was 0.6 days (range 0–14 days). Most patients presented with a history of fever (140/151, 93%) and respiratory symptoms (123/151, 82%). Gastrointestinal symptoms (64/151, 42%), rash (32/151, 21%) and headache (13/151, 9%) were less frequently reported. The majority of cases (98/151, 65%) presented with a simple febrile seizure, including most of the cohort of children with a past history of febrile seizures (27/38, 71%). Seizures were complex in 50/151 (33%) cases: prolonged seizures (lasting 18–60 min) occurred in 17/151 (11%), multiple seizures (2–10 seizures) in 38/151 (25%) and focal seizures in 12/151 (8%), with some overlap between these groups. Insufficient information was recorded in 3/151 (2%) to determine whether they had simple or complex febrile seizures.

Children with complex febrile seizures were younger than those with simple febrile seizures (median 1.4 years vs 1.8 years). Anticonvulsant medications, used to terminate seizures in 19/151 (13%), were more often administered to patients with complex compared to those with simple febrile seizures (14/50, 28% vs 5/98, 5%; *p* < 0.001). For those requiring admission, the median length of stay was 1 day (range 0–8 days). Admissions were more common for complex compared to simple febrile seizures (37/50, 74% vs 32/98, 33%; *p* < 0.001). No patients were admitted to the intensive care unit and all patients were reported to have had a complete neurological recovery.

Virological testing was successfully carried out on per-nasal samples in 143/151 (95%) cases. At least one virus was identified in 102/143 (71%) and viral co-infection in 48/143 (34%). In children with a previous history of febrile seizures, at least one virus was detected in 31/36 (86%). Rhinovirus, Adenovirus and Enterovirus were the most commonly encountered viruses (31/143, 22%; 30/143, 21%; 28/143, 20%; respectively), but were frequently identified in the presence of other viruses (Table 1). Influenza was identified in 19/151 (13%) and usually as a single viral infection. The proportion of cases presenting with complex febrile seizures were highest in the cohorts with influenza and HHV-6 (42 and 41% respectively), but differences from other groups did not reach statistical significance. Most influenza cases (16/19, 84%) presented during the Australian influenza season (May-October). The same seasonal distribution was observed across the whole enrolled cohort (125/151, 83%). Per-rectal samples were analysed in 58/151 (38%) but did not reveal any viruses that were not also identified on corresponding per-nasal samples. No cases of Rotavirus infection were found. No pathogen specific differences in clinical presentation and admission were identified (Table 2).

**Table 1:** Viral pathogens identified in isolation and in co-infection

Virus identified	Single infection	Viral co-infection	Total cases (*n* = 143)
Rhinovirus	6/31 (19%)	25/31 (81%)	31 (22%)
- RV-A			18
- RV-C			13
Adenovirus	6/30 (20%)	24/30 (80%)	30 (21%)
Enterovirus	12/29 (41%)	16/28 (57%)	28 (20%)
- Coxsackie A2			2
- Coxsackie A4			5
- Coxsackie A5			2
- Coxsackie A6			5
- Coxsackie A9			1
- Coxsackie A14			1
- Coxsackie B1			2
- Coxsackie B2			2
- Cosxackie B4			1
- Echo6			2
- Echo9			1
- Echo14			1
- Echo19			1
- Echo30			1
Influenza	11/19 (58%)	8/19 (42%)	19 (13%)
- A/H3N2			13
- A/H1N1			0
- B			6
Human Herpesvirus 6	3/17 (18%)	14/17 (82%)	17 (12%)
Coronavirus	2/13 (15%)	11/13 (85%)	13 (9%)
- HCOV-OC43			8
- HCOV-NL63			2
- HCOV-HKU1			2
- HCOV0HL63			1
Respiratory Syncytial Virus	6/13 (46%)	7/13 (54%)	13 (9%)
- RSVA			8
- RSVB			3
Human Bocavirus	2/13 (15%)	11/13 (85%)	13 (9%)
Parainfluenza III	4/7 (57%)	3/7 (43%)	7 (5%)
Human metapneumovirus	2/5 (40%)	3/5 (60%)	5 (3%)

**Table 2:** Impact of viral pathogens on clinical presentation and management

	Simple FS	Complex FS	Anticonvulsant given	Admission
No virus (*n* = 41)	28 (68%)	13 (32%)	6 (15%)	19 (46%)
One virus (*n* = 54)	36 (67%)	17 (31%)	5 (9%)	21 (39%)
Two or more viruses (*n* = 48)	30 (63%)	16 (33%)	7 (15%)	27 (56%)
Rhinovirus (*n* = 31)	19 (61%)	11 (35%)	5 (16%)	14 (45%)
Adenovirus (*n* = 30)	21 (70%)	7 (23%)	3 (10%)	16 (53%)
Enterovirus (*n* = 28)	19 (68%)	9 (32%)	1 (4%)	14 (50%)
Influenza (*n* = 19)	9 (47%)	8 (42%)	1 (5%)	8 (42%)
HHV6 (*n* = 17)	10 (59%)	7 (41%)	2 (12%)	10 (59%)

Additional requested diagnostic investigations included urine culture (36/151; 24%), blood culture (44/151; 29%) and cerebrospinal fluid (CSF) analysis (9/151; 6%). Urine and CSF cultures were negative in every case. Of four cases with positive blood cultures, three were considered contaminants; one was positive for *Streptococcus pneumoniae*. This case also had multiple viruses identified on per-nasal sample (Adenovirus, HBoV, HHV6, HMPV, Enterovirus A-16, Rhinovirus A).

Sixteen subjects (11%) presented within 14 days of routine childhood vaccinations, mostly with simple febrile seizures (Table 3). Of these, 14/16 (88%) had been administered measles, mumps and rubella (MMR) containing vaccines a median of 9 days prior to their febrile seizure (3 had been administered the measles, mumps, rubella, varicella (MMR-V) vaccine); 9/16 (56%) also had at least one virus identified. One child had a febrile seizure 2 days after administration of routine 6-month immunisations (Infanrix-hexa, Prevenar-13) and another presented 9 days after Varicella zoster virus vaccination; in both cases at least one virus was also identified. None of the children enrolled in the study had received an influenza vaccination.

**Table 3:** Impact of recent immunization on clinical presentation and management

	Simple FS	Complex FS	Anticonvulsant given	Admission
Any vaccine (*n* = 16)	13 (81%)	3 (19%)	1 (6%)	8 (50%)
MMR (*n* = 14)	11 (79%)	3 (21%)	1 (7%)	7 (50%)
Vaccine plus virus detected (*n* = 9)	8 (89%)	1 (11%)	0	3 (33%)
No vaccine (*n* = 135)	85 (63%)	47 (35%)	18 (13%)	64 (47%)

## Discussion

These data demonstrate that, despite increasing interest in vaccination as a cause of febrile seizures in young children, respiratory viral infections are more commonly found in children with febrile seizures than a history of recent vaccination. Viruses were frequently detected in the nasopharynx of children with febrile seizures, but rectal sampling did not provide any additional information. Respiratory viruses such as influenza, rhinovirus and adenovirus were frequently detected. Enterovirus was also commonly detected, although no one strain was predominant. Viral coinfection was common and is indicative of the tendency of children aged 6 months to 5 years to acquire repeated viral respiratory tract infections and demonstrate prolonged viral shedding. Whether or not coinfection is important in the pathogenesis of fever in children with febrile seizures is not clear from these data. In contrast to other viruses found in this study, the majority of influenza and parainfluenza positive cases had only the one pathogen detected. Influenza is known to have an important association with febrile seizures [4, 5, 7–9], with a recent study reporting febrile seizures complicating influenza in 4% of children aged less than 5 years, admitted with influenza in the United States [24].

Clustering of febrile seizures in the 2 weeks following MMR containing vaccines is an expected finding, given previous studies which demonstrate an association, with the greatest risk occurring between day 5 and day 12 following vaccination [10, 13–16]. It is not possible to determine causality in this study though, especially given that half of the subjects with febrile seizure following MMR also had at least one virus identified. The lack of detection of febrile seizures following other childhood vaccines is reassuring. Current influenza vaccines in use for children aged less than 5 years have demonstrated excellent safety profiles [25]. The Australian Childhood Immunisation Register recorded more than 28,000 doses of trivalent influenza vaccine administered to children aged 6 months to 5 years in Western Australia during the study period; yet no children were enrolled in this study with febrile seizure following influenza vaccine.

The study had several limitations. Firstly, enrollment was skewed towards children who were admitted as inpatients, due to increased opportunities for study personnel to access children and their caregiver(s) prior to discharge from hospital. The high proportion of admitted children is reflected in higher than expected rates of complex febrile seizures and use of anticonvulsant therapy in acute management. It is possible that this enrollment bias may have impacted on the range of virologic diagnoses, although the fact that viral etiology was similar in simple and complex febrile seizures suggests that the impact of this potential bias was small. The exclusion of children with central nervous system (CNS) infections and/or abnormal CSF findings enabled the study to describe the findings of true febrile seizures, but it also precluded any comparison of clinical features between children with CNS infections and those with febrile seizures where CNS infection has not been confirmed or suspected. In addition, the lack of a control group limits the ability to attribute fever (or febrile seizures) to viruses that were detected in the nasopharynx. Previous PCR studies have reported high rates of virus detection in the nasopharynxes of healthy, asymptomatic children [26, 27] and it is possible that some children enrolled in this study were colonized rather than infected with detected organisms.

## Conclusion

Respiratory viruses are important in the etiology of fever in children who present with febrile seizures. Administration of vaccines in the 2 weeks prior to febrile seizures was infrequently reported in our series. Given the large number of vaccines administered to children in WA during the study period, this finding suggests that immunization is not a common cause of seizures in our setting.

## Abbreviations

CNS: Central nervous system
CSF: Cerebrospinal fluid
ED: Emergency Department
FEVER: Febrile Seizures: Viruses and their Etiologic Role
HBoV: Human bocavirus
HHV6: Human Herpes Virus 6
HMPV: Human metapneumovirus
MMR: Measles mumps rubella
MMR-V: Measles mumps rubella varicella
NPA: Nasopharyngeal aspirate
PCR: Polymerase chain reaction
RSV: Respiratory syncytial virus
WA: Western Australia
